# A qualitative study of community perception and acceptance of biological larviciding for malaria mosquito control in rural Burkina Faso

**DOI:** 10.1186/s12889-018-5299-7

**Published:** 2018-03-23

**Authors:** Peter Dambach, Margarida Mendes Jorge, Issouf Traoré, Revati Phalkey, Hélène Sawadogo, Pascal Zabré, Moubassira Kagoné, Ali Sié, Rainer Sauerborn, Norbert Becker, Claudia Beiersmann

**Affiliations:** 1 Institute of Public Health, University of Heidelberg, Im Neuenheimer Feld, 324 Heidelberg, Germany; 2 Centre de Recherche en Santé de Nouna, Nouna, Burkina Faso; 3 Epidemiology and Public Health Division, University of Nottingham, Nottingham, UK; 4 German Mosquito Control Association (KABS), Speyer, Germany; 5 Centre for Organismal Studies, University of Heidelberg, Heidelberg, Germany

**Keywords:** *Bacillus thuringiensis* Israelensis, Vector control, Community acceptance, Perception, Qualitative study, West Africa

## Abstract

**Background:**

Vector and malaria parasite’s rising resistance against pyrethroid-impregnated bed nets and antimalarial drugs highlight the need for additional control measures. Larviciding against malaria vectors is experiencing a renaissance with the availability of environmentally friendly and target species-specific larvicides. In this study, we analyse the perception and acceptability of spraying surface water collections with the biological larvicide *Bacillus thuringiensis israelensis* in a single health district in Burkina Faso.

**Methods:**

A total of 12 focus group discussions and 12 key informant interviews were performed in 10 rural villages provided with coverage of various larvicide treatments (all breeding sites treated, the most productive breeding sites treated, and untreated control).

**Results:**

Respondents’ knowledge about the major risk factors for malaria transmission was generally good. Most interviewees stated they performed personal protective measures against vector mosquitoes including the use of bed nets and sometimes mosquito coils and traditional repellents. The acceptance of larviciding in and around the villages was high and the majority of respondents reported a relief in mosquito nuisance and malarial episodes. There was high interest in the project and demand for future continuation.

**Conclusion:**

This study showed that larviciding interventions received positive resonance from the population. People showed a willingness to be involved and financially support the program. The positive environment with high acceptance for larviciding programs would facilitate routine implementation. An essential factor for the future success of such programs would be inclusion in regional or national malaria control guidelines.

**Electronic supplementary material:**

The online version of this article (10.1186/s12889-018-5299-7) contains supplementary material, which is available to authorized users.

## Background

Although considerable success in the fight against malaria has been achieved in the last decade, it still remains a major public health challenge in sub-Saharan Africa. In particular, many West African countries have not been able to achieve the rigorous reduction of new malaria cases compared to East African countries [1]. Today, routine vector control for sub-Saharan Africa is predominantly focused on adult mosquitoes. The first-line vector-control intervention is long-lasting insecticidal nets (LLINs), followed by indoor residual spraying (IRS) for selected settings. Those methods have proven to be highly effective, but challenges arise from mosquito resistance to insecticides [2–5] and the increasing presence of mosquitoes that feed and rest outdoors [6].

An additional pillar for vector control is upstream in the biological development of vector mosquitoes and targets mosquitoes in their larval stages. All actions targeting vector larvae in their breeding sites are subsumed under the term ‘larval source management’ (LSM). One LSM approach is larviciding—the use of chemical or biological agents to kill mosquito larvae when administered in breeding sites. Larviciding against malaria has received little attention over the last 60 years, mostly due to the devastating environmental effects of DDT discovered in the 1950s [7]. However, we now have an entirely new class of biological larvicides, *Bacillus thuringiensis israelensis* (*Bti)* and Bacillus sphaericus (*Bs*), which are proven to be environmentally sound, easy to apply and broadly available. Their mode of action almost uniquely targets the most common disease vector mosquitoes such as *Anopheles*, *Aedes* and *Culex*, while leaving other genera unaffected and shows no (*Bti*) or little (*Bs*) resistance [8, 9]. The control of *Anopheles* larvae with *Bti* has been shown to be effective in several small and large field trials in urban and rural environments [10–13]. Larviciding is not influenced by personal behaviour (e.g. sleeping under a bed net from dusk to dawn) but is deployed at the community level. Cost estimations of several studies in Africa indicate that larviciding is not only cost-effective, but also cost-competitive with other malaria control strategies [13–15]. In contrast to LLINs and IRS, environmental larviciding requires the consent and cooperation of the village/community at large.

Gathering information on people’s knowledge about, attitude towards, and perception of the success of larviciding programs is vital to determine how the programs should be structured and implemented. Only a few studies have been published on the perception of larviciding within African communities [16, 17]. Furthermore, existing research has not yet evaluated whether or not communities perceive larviciding as successful, but instead has focused on people’s knowledge and attitude towards it. The perceived success of lowering the malaria burden and vector nuisance may be a crucial determinant of whether or not an intervention program receives community support, irrespective of epidemiological or entomological data. The objectives of this qualitative study using in-depth interviews (II) and focus group discussions (FGD) were to evaluate people’s perception of the success of *Bti*-based larviciding for malaria control and assess its acceptance by local communities. Additionally, we researched the community’s trust and confidence in such a program.

## Methods

### Study area

This study took place in the Nouna health district (NHD) in the Kossi province in North-western Burkina Faso and included 127 rural villages ranging from several hundred to a few thousand inhabitants who mostly engage in subsistence farming. Within the health district, there are 13 rural health centres (CSPS) delivering basic medical treatment in a catchment area of up to 25 km [18]. The region is characterized as dry savannah with a sub-Saharan climate, and an approximately 800 mm annual mean precipitation and 27.8 °C mean temperature. The region features one rainy season that extends from June to September and a dry season that lasts from November to April. During the rainy season, water accumulates in ponds, hoof prints, traditional brickworks and partially wet rice fields. These serve as mosquito breeding sites, while during the dry season only few water bodies remain. Main vectors for malaria (> 98%) are *Anopheles gambiae* s.l. and to a much smaller extent, *A. funestus* and *A. nili* [19]. Malaria transmission occurs throughout the year with a marked seasonal peak during the late rainy season in August. The entomological inoculation rate, which is defined as the number of infective bites per person per year, has been reported to be as high as several hundred [20, 21]. The national malaria control program includes the use of insecticide treated nets (ITNs), intermittent preventive treatment for pregnant women, and early diagnosis through fever and rapid diagnostic testing. Indoor residual spraying (IRS) and larviciding have not been implemented to date.

### Study design

This qualitative study was part of a larger EMIRA project (Ecologic Malaria Reduction for Africa) conducted in collaboration with the Nouna Health Research Center (NHRC). It was a community-based intervention trial using *Bti* as a larvicide, and examined the added health benefit of biological larviciding in rural Burkina Faso. Methods have been described in detail elsewhere [22–24]. In brief, each of the 127 NHD rural villages were assigned to one of three study arms: *Bti*-based LSM in all breeding sites within and around villages (100% treatment arm); selective treatment of breeding sites with the assumed highest vector larvae productivity (50% treatment arm) [25, 26] and an untreated control group. Each study arm was distributed across three geographically separated clusters to account for possible differences in mosquito ecology within the survey region. Clustering the villages into distinct treatment and control areas took into consideration the mosquitoes´ flight range of several kilometres [27, 28]; therefore, randomization on a village level was not possible. Nouna, the semi-urban district capital, received only full *Bti*-based LSM, since assigning the town quarters to distinct study arms was not possible due to mosquito flight ranges and human movements.

### Sampling and data collection

**Fig. 1:**
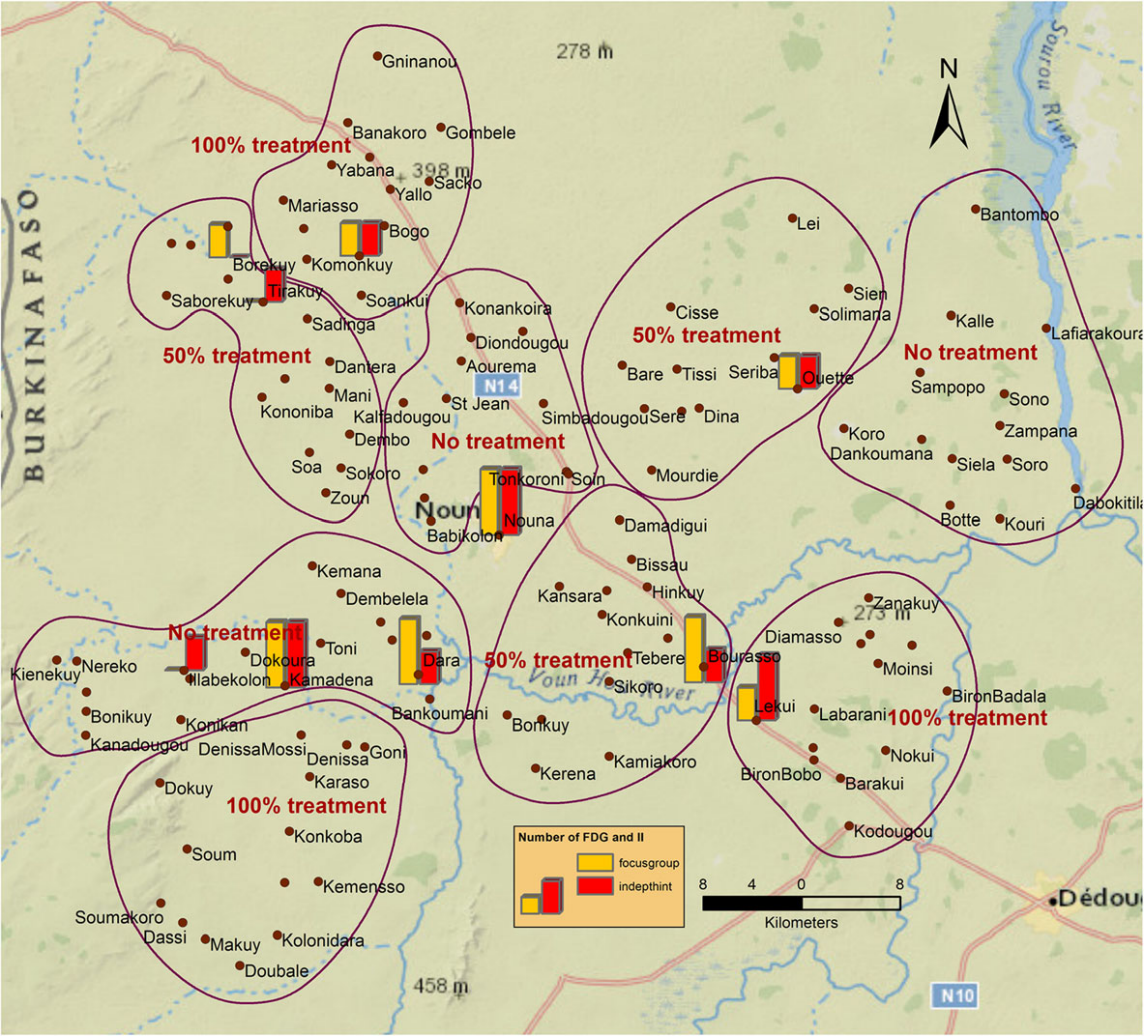
Study area with intervention and control clusters. Bars indicate villages in which FGD (yellow) and II (red) were performed; bar heights indicate their number (one or two)

For this qualitative study, data were collected from 12 FGDs and 12 IIs (Fig. 1). Convenient sampling was applied for the FGDs, four FGDs have been conducted within each study arm. The villages were informed of the FGDs and IIs prior to the study and the day of data collection. All villagers were eligible to join the FGDs and number of participants ranged from 4 to 11. For the II, persons in key positions in the community, such as imams, teachers and village leaders were identified with the help of community informants of the NHRC and through snowball sampling. Those people generally have an above average school education and interacted with large parts of the village population on a daily basis. Therefore, the II participants were well informed about the villagers’ perception of the mosquito control program. The FGDs lasted on average about 1 h30 and the IIs about one hour, and both were voice recorded with participants ‘consent. NHRC-trained field personnel performed the data collection and transcription. PD developed the interview guide in collaboration with NHRC colleagues in French (Additional file 1). The data collection was performed in French and was supported by explaining in the local lingua franca Dioula when needed.

### Data analysis

The NVivo software package (NVivo Pro Version 11) and Excel (Microsoft Excel 2010) were used for data analysis. MMJ and CB (both fluent in French) performed a content analysis on all transcripts using a mix of deductive and inductive coding. Randomly selected transcripts were coded by both authors and discrepancies crosschecked and discussed to obtain a clear coding scheme.

## Results

Results are presented on: (1) community knowledge, attitudes, and practices regarding malaria (i.e. perceptions of malaria, knowledge and perceptions of mosquito development, practices of malaria prevention and control, and opinions on the reduction of mosquitoes and malaria cases) and (2) knowledge, perceptions and acceptability of the mosquito control program (i.e. knowledge of the existence of the program, opinions on the program, suggestions on how to improve the program, knowledge and opinions on the larvicide, knowledge of malaria and ITN use after the program, and willingness to pay).

### Knowledge, attitudes, and practices regarding malaria

#### Malaria perceptions

Almost all respondents acknowledged mosquitoes were transmitting malaria.

*"It is through the mosquitoes that we catch malaria." (FGD 1, woman, 100% intervention)*

However, many respondents cited other modes of malaria transmission such as dirt and dust in many forms (e.g. dirty food, dirty environment, or eating with dirty hands). Water was also referred by many participants, especially dirty and/or stagnant water and mosquitoes which drank and/or transported dirty water.

*"If the mosquitoes drink dirty water and they bite you they can transmit malaria." (FGD 6, man, no intervention)*

Certain weather conditions (e.g. cold, wind, or rain) were also cited by many interviewees as a cause for malaria. A small number of respondents believed that malaria could be transmitted directly from other humans, e.g. through cough or vomit.

### Knowledge and perceptions of mosquito development

The study communities had knowledge on how mosquitoes develop. The vast majority of respondents linked mosquito development with water and generally, this water was referred to as “dirty” or/and “stagnant”. Some participants also mentioned the rainy season, rain itself, and swamps as responsible for mosquito development.

Many respondents stated that the mosquitoes develop from eggs.

*“Mosquitoes are born from used waters, edges of swamps, loopholes, toilets, places that contain the animals’ excrement and are always dirty in the rainy season. Here is where mosquitoes lay eggs. They hatch and mosquitoes come out, ah! This is why mosquitoes become numerous to give us diseases.” (FGD 11, 50% intervention, man)*

Larvae were also mentioned in connection with mosquito development. In the view of many respondents, a dirty environment (e.g., dirty water, toilets, plants, dirt or waste in general), favours the development of eggs, larvae, and mosquitoes. Many participants mentioned swamps as the main location where larvae and/or eggs could be found.

*“If there are dirty waters, they lay eggs in it, and also in the garbage that is rotting, so the eggs will spark out to give birth to mosquitoes.” (FGD 1, 100% intervention, woman)*

*“Mosquitoes lay their eggs in water, become larvae and become numerous. Then they come to bite us.” (FGD 9, 50% intervention, man)*

*“They are found on the swamps’ edge. Where water is frequently found, the eggs are there.” (II 8, 50% intervention, man).*

Other localities referred to included: the trash, places with no light or dirty, plants, herbs or foliage, in nature in general, inside houses, on beds, toilet and stagnant waters, wells and gutters, humid places and inside shea nuts.

*“(...) if there's grass around your compound, it's over there that the mosquitoes are born.” (FGD 7, No intervention, woman)*

*“During the winter, the women will pick up the shea nuts and bring them in their courtyards to let them rot, they are nests for mosquitoes, this is not at all good.” (FGD 3, 100% intervention, man)*

The big majority had an idea about mosquitoes’ development, stages and favourable conditions. Respondents rarely said that they didn’t know how mosquitoes evolve.

*“We do not understand this, we were born and the mosquitoes were there, and we call them mosquitoes, otherwise saying that something else turns into a mosquito, we do not know that.” (FGD 7, No intervention, woman)*

### Practices for malaria prevention and control

Interviewees stated that the main method to protect against malaria was by sleeping under an insecticide treated net (ITN) and recommended that children and adults should use them. However, some expressed that only children should sleep under the ITN. Other frequently mentioned malaria prevention and mosquito control methods as: keeping the living environment clean, having a clean house/courtyard as well as safe food and clothes, and evacuating waste. The use of indoor and outdoor spraying and burning mosquito coils were also important.

*“You only need to protect yourself by sleeping under the mosquito nets and lighting the mosquito coils.” (FGD 3, 100% intervention, man)*

*“To avoid malaria, it is necessary to fight against dirt, it is necessary to evacuate the waste/used water of the backyard, protect meals/nutrition, to wash hands well before eating, to take care of your children, to make them sleep always under an impregnated mosquito nets.” (II 12, 100% intervention, woman)*

Other contributions to malaria prevention and mosquito control included covering toilets and latrines, cutting plants around the house, keeping the animals away from the house, using repellents, washing hands before eating, using shoes during the rainy season or using shea nut cream inside nostrils to prevent colds.

The most cited method to control mosquito larvae specifically was chemical treatment and spraying of water ponds, as well as other locations including house courts or toilets.

*“You have to take products to put in our swamps to destroy the mosquito larvae.” (FGD 10, 100% intervention, woman)*

Once again, leading a “clean” life and keeping the environment or household clean were also frequently mentioned. Participants stated that one should keep toilets clean, evacuate dirty water, and remove herbs and plants from the (house) courts. Additional highlighted strategies mentioned occasionally were e.g. covering pits from pit latrines, pay attention to closing pots with food, and close and fill holes in the ground to avoid stagnant water.

*“In my opinion, to reduce the development of larvae, we have to evacuate the dirty water from the concession, clean our living environment, dig pits for our toilets, where the water will stay, instead of the toilet water will flow at the feet of our walls.” (FGD 5, 50% intervention, man)*

On rare occasions, people pointed to the fact that if all mosquitoes were killed, there would be no larvae, and the challenge is to catch every single larva through spraying/treating water ponds. So, from that perspective, unless there was a total larvae kill, mosquitoes would come anyway.

### Opinions on the reduction of mosquitoes and malaria cases

The majority of respondents in the 100% intervention arm were of the opinion that mosquitoes had decreased.

*“We realized that there are less mosquitoes now than before because of the spraying activity that is done. The application of larvicides was a very good thing.” (FGD 1, 100% intervention, woman)*

*“We realized that the malaria cases in children were not as many as before.” (FGD 1, 100% intervention, woman)*

However, there were also participants with opposing views who saw no effect of the 100% intervention on mosquito density.

*“In my view it was a good initiative but it has not managed to reduce the presence of mosquitoes.” (FGD 3, 100% intervention, man)*

*“In my opinion there was no change, during the rainy season we have many malaria cases.” (FGD 3, 100% intervention, man)*

In the 50% intervention arm, opinions on the effect of the intervention on mosquito density were mixed. Many respondents stated that there had been a reduction of mosquito density and almost all participants acknowledged a general reduction on malaria cases and their severity before and after the intervention.

*“Yes, there were changes; we saw a reduction in mosquitoes.” (FGD 5, 50% intervention, man)*

*“Before we had many malaria cases because of the mosquitoes, we had severe malaria cases that made the children anaemic; but all that has decreased.” (FGD 5, 50% intervention, man)*

However, a small group stated that there had been an increase in malaria cases and severity, thus making malaria more difficult to treat with current medicines and provoking more severe anaemia cases compared to before the mosquito control intervention.

*“Me, I can say there was no change. Before, we saw the mosquitoes only during the rainy season. Now, even in dry season there are mosquitoes. Proof is that the people still sleep under their mosquito nets. The mosquitoes are present in the spraying season and in the dry season.” (FGD 9, 50% intervention, man)*

*“Since the spraying activity started, malaria has become anaemic, severe with unconsciousness and convulsions in the body. Actually, the forms of malaria became numerous.” (FGD 9, 50% intervention, man)*

A few participants mentioned there had been no change in the number of malaria cases after the mosquito control intervention.

*“We did not realize a change, actually.” (FGD 12, 50% intervention, woman)*

### Knowledge, perceptions and acceptability of the program

#### Knowledge of the existence of the program

The vast majority of the participants knew about the larvicide spraying intervention. Most reported having seen the program workers or had even talked to them.

*“Yes, I'm aware of that. They walk around spraying the swamps during the rainy season.” (FGD 11, 50% intervention, man)*

Almost all respondents stated that they learned about the program because they talked to the spraying agents or saw them working. Very few mentioned having been informed by the rural health facilities (CSPS) or CSPS agents.

*“I saw them spraying the swamps and I asked them what were they doing, they told me it was to kill the mosquito larvae.” (FGD 1, 100% intervention, woman)*

The reduction of mosquito eggs, larvae and mosquitoes were perceived to be the main objectives of the program.

*“For this I will speak about those you have chosen to spray water in the village, they spray larvicides in dirty waters and this can help to reduce mosquito larvae.” (II 4, 50% intervention, man)*

### Opinions on the program

The great majority of respondents stated that they perceived the intervention as positive. Many participants valued the spraying program as an effective strategy to control malaria and/or reduce mosquitoes.

*"Before, during the winter, our children cried at night because of mosquitoes (laughs). We could not sleep, but nowadays everyone is sleeping peacefully." (FGD 5, 50% intervention, man)*

*“Here, it looks like there has been a little change. You do not get malaria easily like before, the mosquitoes, which were too much, decreased a bit.” (II 6, 100% intervention, man)*

The intervention was perceived as a support for the communities.

*“I think the community appreciates the activity very much, and everyone recognizes that any initiative to eliminate mosquitoes is a good thing for us, because it's sort of a fight against malaria.” (FGD 3, 100% intervention, man)*

However, a few respondents had other views about the intervention. They mentioned the product was not effective in times of heavy rains, and referred to mosquito populations as remaining constant or even increasing. These participants doubted the effect of spraying on mosquitoes and added that relying only on one control method (spraying) might not be enough. Negative views were more often expressed in the 50% arm of the intervention.

*“We said that the activity is good because it has reduced the mosquitos’ presence. But often when the rains are abundant one cannot feel the effect of the product, which is why others said that it has no effect. Whatever people think, we felt a reduction in the presence of mosquitoes.” (FGD 1, 100% intervention, woman)*

*“In my opinion, as they sprayed, the mosquitoes would die, but they spray and the mosquitoes still do not die, that's what we do not understand. I really do not understand this, it should be able to kill the mosquitoes, but they do not die, if it is night we cannot sleep, I really did not understand this point.” (FGD 12, 50% intervention, woman)*

### How to improve the program

Many respondents wished that the project would be continued and even be reinforced through options such as reducing the time periods between spraying activities and enlarging the spraying activities to regions where the intervention had not yet taken place. Toilets, houses, and compounds were the most frequently mentioned places to add to the intervention.

*“The activity was appreciated by the people; their wish is that you have the means to continue the activity in the years to come.” (II 12, 100% intervention, woman)*

*“In my opinion, the community appreciated the activity very much, but the problem was that not all the village waters were sprayed; it seems that half the water points were sprayed. So the wish of the populations is that spraying is extended to the whole village and also the toilets´ waters.” (FGD 5, 50% intervention, man)*

In the view of the respondents, the program could also be improved through increasing the number of spraying events separated by shorter periods of time. Several participants considered it also important to increase the number of spraying pumps, staff, and staff salaries. Furthermore, they recommended that the work be during the whole year and for a longer period of time.

*“In my opinion it is necessary to continue the spraying but to reduce the interval between spraying sessions.” (FGD 2, 100% intervention, woman)*

*“We want the spraying to continue, the agents have to be well motivated to do the job, if we can increase their salary it would be a good thing.” (FGD 2, 100% intervention, woman)*

A small number of participants said that the larvicide, as a product, should be improved and a few suggested that the product should be changed.

*“Spraying is good if new practices are applied. Even if the larvae are killed and the mosquitoes remain, the eggs will still be laid. We heard that the mosquito egg stage is 7 days. The best is to apply the products that kill eggs, larvae and mosquitoes.” (FGD 11, 50% intervention, man)*

A minority of respondents believed there was a need for awareness and/or explanation sessions with community members as a way to let the population know more about the mosquito control program and win their trust and motivation to cooperate.

*“As others have understood and some not, it is necessary first that the sensitization/awareness is sufficient, then that the choice of the people for this activity is known in advance and that the spraying program is respected.” (II 11, 50% intervention, man)*

Some participants also requested malaria control products such as ITNs, larvicides, and antimalarial treatment for their own use.

*“We must continue spraying but if we could also distribute mosquito nets it would be good.” (FGD 2, 100% intervention, woman)*

A minority of the participants believed the program did not need improvement and should continue as it was.

### Knowledge of and opinions on the larvicide

The majority of the participants declared they did not know anything about the larvicide product itself.

*“We do not know the larvicide.” (FGD 1, 100% intervention, woman)*

A small number of respondents seemed to have seen the larvicide and some even described it having a consistency of peanut paste while others said it was a powder. The larvicide type and its mixing with water was not known by almost all, but it was described by one participant.

*“It is in powder, the colour resembles that of peanut paste.” (FGD 11, 50% intervention, man)*

*“I have already seen it, it is a powder product that is put in water, I have already attended mixing and I observed.” (FGD 11, 50% intervention, man)*

The majority of the respondents considered the larvicide to be safe for humans and animals. A few respondents stated they did not know about the safety of the larvicide and a small minority believed the product was not safe for animals or humans.

*“I inquired and was reassured that the larvicide was harmless to humans and animals. It's only to fight the mosquitoes.” (FGD 3, 100% intervention, man)*

*“What can kill God's small creatures can also kill humans because there's fun in it (laughter).” (II 7, 100% intervention, man)*

When asked, almost all participants answered positively about whether or not they would give permission to spray mosquito breeding places near their houses. A small number of participants would accept it only if the reasons for spraying were adequately explained to them. Only a few respondents would not give permission, and the reasons were related to being careful with spraying near drinking water points, for example.

*“If the larvicide does not kill our animals, our poultry. Only mosquitoes, we will accept so that the mosquitoes are eradicated from our country.” (FGD 11, 50% intervention, man)*

*“The water points where our animals like to drink frequently, we will not give permission to spray them because we never know, if the product is really innocuous.” (FGD 9, 50% intervention, man)*

### Knowledge of malaria and ITN use after the program

Overall, participants stated there was more information and knowledge on malaria transmission after the intervention program, yet this was not always specified. Respondents who did mention this specifically, highlighted having gained more knowledge on mosquitoes as the cause of malaria, as well as the need for mosquito control and prevention methods like not sleeping outside the night, sleeping under an ITN, and emptying stagnant water in gardens.

*"When we asked the spraying agents what they were doing, and they told us that it was to eliminate the mosquitoes. So, during the conversations we understood that the mosquitoes are the basis of the malaria cases we have. It's like a fight against malaria" (FGD 2, 100% intervention, woman)*

#### A few critical responses

*"Since the beginning of the spraying, we have not understood anything about malaria. Until now, the mosquitoes are still there, and bite people. If you go to the CSPS they say that the mosquitoes are the cause of malaria, and because of that there is the spraying. So we have not understood anything of this. There hasn’t been any change." (FGD 12, 50% intervention, woman)*

The majority of respondents did not report any behaviour change regarding the use of ITNs.

*"We continue to sleep under mosquito nets despite the mosquito reduction." (II 12, 100% intervention, woman)*

However, some respondents declared that there was an increase of ITN use after the program, and a similar amount of responses declared to receive for the first time an ITN through the implemented program.

*"Before (long time/before project start) we didn’t know the existence of a mosquito net and there was no spraying also. We went to the bush to cut some things and bring them to our homes. But now we've been helped with that, that's good, and we're sleeping under that (mosquito net). " (FGD 12, 50% intervention, woman)*

A very small number of participants stated that they perceived a reduction in the number of nights sleeping under an ITN.

### Willingness to pay

Respondents were of the opinion that the population could contribute financially to the program.

*“Everyone should agree to give a contribution to help the project.” (II 12, 100% intervention, woman)*

When asked what they would be willing to pay per year for spraying, the participants established a range between 100 and 6000 FCFA for the 50% intervention group and between 100 and 5000 FCFA in the 100% intervention group. The West African FCFA is pegged to the Euro; 100 FCFA equal 0.15 Euro.

Specifically, most for interviewees in the 50% intervention arm declared they were willing to contribute to the implementation of the larvicide intervention with 500 FCFA per year. Some participants stated they were willing to pay 1000 FCFA per year and a very few participants mentioned 100, 200 and 2000 FCFA as their possible contribution.

*“My voluntary contribution per year can be 500 FCFA.” (FGD 5, 50% intervention, man)*

In the 100% intervention arm, specifically, a great number of participants regarded 500 FCFA or 1000 FCFA per year as the amount that they felt comfortable paying voluntarily. Some were willing to pay 200 or 250 FCFA per year, and a few stated 100 FCFA, 2000 FCFA and 10,000 FCFA as their voluntary contribution.

*“I can contribute 1000 FCFA without a problem.” (FGD 10, 100% intervention, women)*

## Discussion

### Malaria knowledge

Generally, there was good knowledge about how malaria is transmitted in the rural study area in Burkina Faso. Mosquitoes were correctly identified as vector for malaria and water was recognised by most respondents as mosquito breeding place. This finding is in line with other studies in which respondents showed similar levels of knowledge on malaria transmission [29–31]. Some participants in this study specified stagnant water and many mentioned dirty or polluted water. This observation might be partially attributable to the fact that people observed high mosquito nuisance near breeding places such as dirty puddles in their courtyards or pit latrines. Many of this study’s participants ascribed increased malaria risk to those places, although in most cases puddles and pit latrines are not suitable for malaria vector breeding due to pollution or temporal stability. That said, dirty puddles and pit latrines are used for larval development by other mosquito genera such as *Culex* and occasionally *Aedes* [32–34]. Since the population was not aware of the various mosquito genera, transmission risk was perceived as general mosquito nuisance, and defined as the abundance of all host-seeking mosquitoes irrespective of species. Furthermore, eggs and larvae were also identified as stages in mosquito development and only a very few participants in this study had no idea or perception of how mosquitoes develop. Since mosquito development and survival is bound to environmental water and humidity, several participants made a logical deduction and described environmental conditions during the rainy season such as cold weather, wind, and inundated areas as triggers for mosquito presence.

### Malaria prevention knowledge and practices

A high number of participants performed practices aimed against vector mosquitoes. Most stated mosquito bed nets were their first choice in personal protection against malaria mosquitoes, particularly during the rainy season. This is congruent with findings from a recent study in the same region [35]. Others trusted mosquito coils and covering pit latrines for mosquito control. Several respondents proposed performing larval source management not only through larviciding, but also via transforming and filling up stagnant water bodies. Nonetheless, vector control practices predominantly included measures against the adult vector and to a lesser extent against vector larvae. Several participants also mentioned less specific methods of mosquito control such as general hygienic precautions including washing hands and not walking without shoes, thus hinting at a less profound understanding of malaria transmission modes probably influenced by awareness campaigns on other diseases.

### Larviciding program knowledge, acceptability and perceived success

The majority of respondents reported having knowledge about the implemented larviciding program from their own observations or talking to spray personnel. Announcements about the project were repeatedly aired over local radio, but this was rarely mentioned as a source of information. This finding might be due to the fact that only few people in the communities possessed a radio and those who owned one might not have listened to it during farming activities.

Although most participants were unfamiliar with the specific larvicide used in the intervention, the program was generally considered as an effective measure to decrease mosquito abundance and to reduce malaria cases. A recent field trial in Rwanda indicated similar results regarding the perceived success in malaria and mosquito nuisance reduction [31]. The product itself was considered to be safe for humans and animals, which was a finding comparable to people’s perception in another study [16]. Almost all participants responded that they would support continuing larviciding activities. There was a high willingness to financially contribute to future larviciding programs [36], which was equally observed in an East African study [16]. It has to be born in mind though that there might be a substantial discrepancy between theoretical and actual willingness to pay.

Very few respondents had negative opinions about the larviciding program, which included mentioning an increase in mosquito numbers. This could be explained by the entomological data from the same study (unpublished data) that showed a reduction of *Anopheles* mosquitoes in all study intervention villages, but an occasional increase in other mosquito genera such as *Culex* and *Aedes*. Most of this increase, however, can be attributed to naturally higher mosquito numbers in the intervention years (2014 and 2015) compared to the baseline (2013). This might explain some participants’ perception of the intervention’s lower success in reducing malaria vectors. Compared to the 100% treatment arm, respondents from the 50% treatment arm expressed more often a limited success of spraying activities regarding mosquito reductions and malaria cases. This observation is in line with results from mosquito catches in light traps which showed 15% to 20% fewer reductions in the 50% treatment arm (Dambach, unpublished data).

### Limitations

There are several limitations to this study. Interviewed individuals and participants of FGD might have perceived community pressure to answer questions on larviciding success in a positive way and pronounce their support for the project. Interviewers and leaders of FGDs are often seen as persons of high rank and respect, in particular when they are connected with the health research centre. Although interviewers tried to emphasize that questions should be answered honestly, a certain level of unconscious intimidation was possible.

### Future research/recommendations

To further increase the perceived success and hence acceptance of future larviciding campaigns, pit latrines and other breeding sites within compounds predominantly infested with *Culex* and *Aedes* mosquitoes should be equally treated. Although a reduction of those mosquito genera does not ameliorate the malarial situation, it will largely reduce mosquito nuisance, which is beneficial to the local communities’ perception of success. As an additional benefit, this would address diseases such as dengue, chikungunya and lymphatic filariasis, that are transmitted by *Aedes* and *Culex* mosquitoes. It is important to develop malaria control activities together with the community, inform them and take their opinions into account when further developing and ameliorating these activities on a community level.

## Conclusions

Our study showed that the performed larviciding intervention resonated positively amongst the population in rural villages and the semi-urban town of Nouna in Burkina Faso. People are generally content with and positively minded towards larviciding programs. Awareness of malaria transmission increased during spray interventions and people showed a willingness to become involved and offer financial support. The population would welcome the routine implementation and continuation of this larviciding program. There was a positive environment with high acceptance for larviciding programs across the study sites, which would facilitate their routine implementation in the future.

## Additional file

Additional file 1: English translation of the interview guide used to direct discussions. The interview guide used in the field is in French language. (DOCX 12 kb)

## Abbreviations

*Bs*: *Bacillus sphaericus*
*Bti*: *Bacillus thuringiensis israelensis*
CSPS: Rural health center
DDT: Dichlordiphenyltrichlorethan
EIR: Entomological Inoculation Rate
EMIRA: Ecologic Malaria Reduction for Africa
FGD: Focus Group Discussion
II: In-depth Interview
IRS: Indoor Residual Spraying
ITN: Insecticide Treated Net
ITTp: Intermittent Preventive Treatment in pregnant women
LLIN: Long Lasting Insecticidal Net
LSM: Larval Source Management
NHD: Nouna Health District
NHRC: Nouna Health Research Center
SSA: Sub-Saharan Africa
