# Supplementary material for: A qualitative study of community perception and acceptance of biological larviciding for malaria mosquito control in rural Burkina Faso
Source: BMC Public Health. 2018 Mar 23;18:399. doi: 10.1186/s12889-018-5299-7 (PMC5865284; doi:10.1186/s12889-018-5299-7)
Supplement: Supplementary file 1 — English translation of the interview guide used to direct discussions. The interview guide used in the field is in French language. (DOCX 12 kb) [file 12889_2018_5299_MOESM1_ESM.docx]

**Interview guide**

**Part I: Knowledge and prevention practices**
How do you think malaria is transmitted?
How do you think mosquitoes are born?
What factors do you think promote mosquito development?
Where do you think mosquito larvae can be found?
What can be done to prevent malaria? / Are each of you going to tell me what they are doing to prevent malaria?
Can reducing mosquitoes help reduce malaria?
What actions do you think can reduce the abundance of mosquitoes?
What measures do you think can reduce the development of mosquito larvae?

**Part II: Knowledge, perception and acceptability of the program**
Do you know that spraying actions have been carried out in this area to eliminate mosquitoes?
Have you seen the spray teams with knapsack sprayers, spraying in this area?
How did you find out about this activity for the first time?
What do you think of this intervention?
What do you think is the community's impression of the activity?
Could you tell us what we could improve for future interventions?
Could you tell us about the changes you noticed after the project was implemented?
Do you have knowledge of larvicides?
Will you give permission to apply larvicides to water points near the house?
Do you think that the larvicide is harmless to humans and animals?
Do you trust that the application of larvicides will reduce the risk of malaria infection?
How do you see the presence of mosquitoes compared to past years?
How do you see the number of malaria cases compared to the past years?
Would you volunteer to contribute to a larviciding application program? if so, with what amount?
You told me that you all sleep under mosquito nets. Did the spraying program alter this behavior?
